# Telemedicine Preparedness Among Older Adults With Chronic Illness: Survey of Primary Care Patients

**DOI:** 10.2196/35028

**Published:** 2022-07-27

**Authors:** Kelsey Ufholz, Amy Sheon, Daksh Bhargava, Goutham Rao

**Affiliations:** 1 Department of Family Medicine and Community Health School of Medicine Case Western Reserve University Cleveland, OH United States; 2 Public Health Innovators LLC Cleveland, OH United States; 3 Department of Family Medicine University Hospitals Cleveland, OH United States

**Keywords:** telemedicine, seniors, primary care, chronic illness, health equities, telehealth, older adult, healthcare, health care, digital health, senior health

## Abstract

**Background:**

Older adults are a high priority for telemedicine given their elevated COVID-19 risk and need for frequent provider contact to manage chronic illnesses. It seems that many older adults now use smartphones but few studies have examined their overall readiness for telemedicine.

**Objective:**

The aim of this study is to survey older primary care patients about their telemedicine preparedness, including internet usage, internet-capable devices, telemedicine experiences and concerns, and perceived barriers. Results were used to inform a telemedicine preparedness training program.

**Methods:**

Community-dwelling older adult patients (aged 65-81 years; N=30) with a chronic health condition that could be managed remotely who were present at a family medicine clinic that primarily serves an urban African American population for a prescheduled in-person appointment were asked to complete a brief survey written for this study. Data were collected February-June 2021 at a large, urban, Midwestern hospital. To minimize patient burden, the survey was limited to 10 questions, focused on the most critical topics.

**Results:**

Most participants (21/30, 70%) reported having a device that could be used for telemedicine and using the internet. However, about half had only a single connected device, and messaging and video calling were the most commonly used applications. Few used email and none used online shopping or banking. Only 7 patients had had telemedicine appointments. Telemedicine users were younger than nonusers and used more internet functions than nonusers. Only 2 people reported problems with their telemedicine visits (technology and privacy). Nearly all respondents recognized avoiding travel and COVID-19 exposure as telemedicine benefits. The most common concerns were loss of the doctor-patient connection and inability to be examined.

**Conclusions:**

Most older adults reported having devices that could be used for telemedicine, but their internet use patterns did not confirm the adequacy of their devices or skills for telemedicine. Doctor-patient conversations could be helpful in addressing telemedicine concerns but device and skill gaps must be addressed as well.

## Introduction

### Background

Since the COVID-19 pandemic began, telemedicine appointments have replaced many in-person health care visits [1,2]. However, older people are less likely to participate in telemedicine, preferring in-person care or foregoing care altogether [3-6]. With a high prevalence of chronic conditions and vulnerability to COVID-19 morbidity and mortality through exposure to others in health care environments [1-4], promoting telemedicine use among older adults should be a high priority.

### Older Adults’ Barriers to Telemedicine

Older adults face significant barriers to participation in telemedicine, including limited access to the internet and devices suitable for telemedicine [7]. Older adults may also lack digital skills or have visual, auditory, and tactile difficulties with telemedicine, or be uncertain about whether or when to use it. To inform our plans for offering telemedicine training to older adults presenting to an outpatient family medicine teaching clinic that serves predominantly African American, economically disadvantaged adults with chronic illness in Cleveland, Ohio, we administered a survey to learn about their telemedicine readiness, and telemedicine barriers and facilitators.

## Methods

### Participants

We sought to recruit 30 participants, the minimum recommended sample size for estimating univariate averages, and a number thought adequate to identify common patient journeys that would guide our plans for telemedicine training [8,9]. Participants were recommended to this convenience sample by primary care providers who were familiar with their medical history and the study criteria. Inclusion criteria included age ≥65 years and having a chronic health condition (diabetes, hypertension, arthritis, etc) that could be managed remotely. Patients with known dementia, residence in a long-term care facility, and presenting with an acute condition requiring in-person care (eg, fall or chronic obstructive pulmonary disease exacerbation) were ineligible.

### Survey Instrument

Because existing surveys tend to lack the specificity needed to determine the adequacy of devices and skills for telemedicine, we designed and pretested a new survey instrument based on a review of the literature, and input from our primary care providers and a digital equity expert (Multimedia Appendix 1). Because we were not offering compensation, we minimized patient burden by limiting the survey to 10 questions. Topics included demographics, experience using telemedicine, problems and perceived barriers, ownership of telemedicine-ready devices(s), and use of various internet functions.

### Procedures

Patients present at an in-person primary care visit for issues that could be accomplished remotely were approached by a research assistant to complete the survey between February and June 2021. Data were collected on paper, with a research assistant available to read the survey questions and record responses if needed. The research assistant entered anonymous responses into a REDCap database to protect patient privacy. Descriptive statistics were calculated to inform our telemedicine readiness training plans. Chi-square tests were used to test for statistical significance, α=.05.

### Ethical Considerations

University Hospitals’ Institutional Review Board determined the study (2021611) to be no more than minimal risk and granted expedited approval. Written informed consent was not required but prior to beginning the study, participants received written information informing them that they were invited to participate in a voluntary research study and were free to decline participation.

## Results

### Devices and Internet Usage

Of 30 respondents, 25 (83%) said they had devices that could be used for a telemedicine visit and that they went on the internet, but just 7 of 30 (23%) had had telemedicine visits. However, few patients had advanced devices (iPhones, desktops, laptops, or tablets) that are best suited to telemedicine. In addition, 14 of 30 respondents (47%) had only a single device that was not an iOS-based mobile device (Table 1) and may have had limited videoconferencing ability. All participants with devices said they used them for “messaging on the internet,” but this was the only function used by 12 of 30 respondents (40%). No one used the internet for banking or shopping, and few used internet functions commonly needed for telemedicine (email: 7 respondents, 23%; video calling: 9 respondents, 30%) (Table 1).

**Table 1:** Survey participant demographics and telemedicine readiness.

Demographics and telemedicine readiness			Participants, n		Participants, %
**Age (years)^a^**					
	65-74	24		80	
	75-80	5		17	
	80-89	1		3	
**Chronic conditions**					
	1	5		17	
	2	13		43	
	3	10		33	
	≥4	2		7	
	Hypertension	19		63	
	Diabetes	18		60	
**Device ownership**					
	iPhone	5		17	
	Desktop, tablet, laptop	6		20	
	Other smartphone only	14		47	
	0	5		17	
	1	21		70	
	≥2	4		13	
**Internet use**					
	Telemedicine visit	7		23	
	Video calls	9		30	
	Entertainment	5		17	
	Email	4		13	
	Messaging only	12		40	
	Work, banking, shopping	0		0	
	No internet functions	5		17	
	1 internet function	12		40	
	2 internet functions	8		27	
	3 internet functions	5		17	
**Telemedicine advantages**					
	No travel	29		97	
	Avoid COVID-19	25		83	
**Telemedicine disadvantages**					
	Doctor cannot examine me	7		23	
	Loss of personal connection	10		33	
	Inferior care quality	4		13	
	Lack of privacy	7		23	
	Other disadvantage	3		10	

^a^Mean age 70.8 (SD 4.3) years; range 65-81 years.

### Telemedicine Experiences and Perceptions

**Figure 1:**
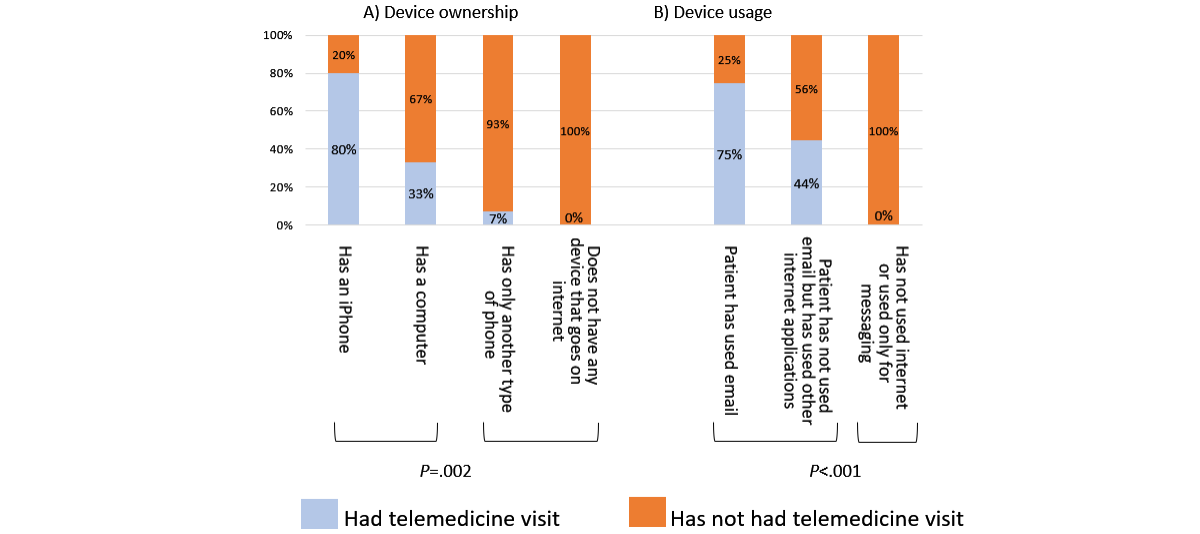
Comparisons of likelihood to have had a telemedicine visit by device ownership and device usage.

**Figure 2:**
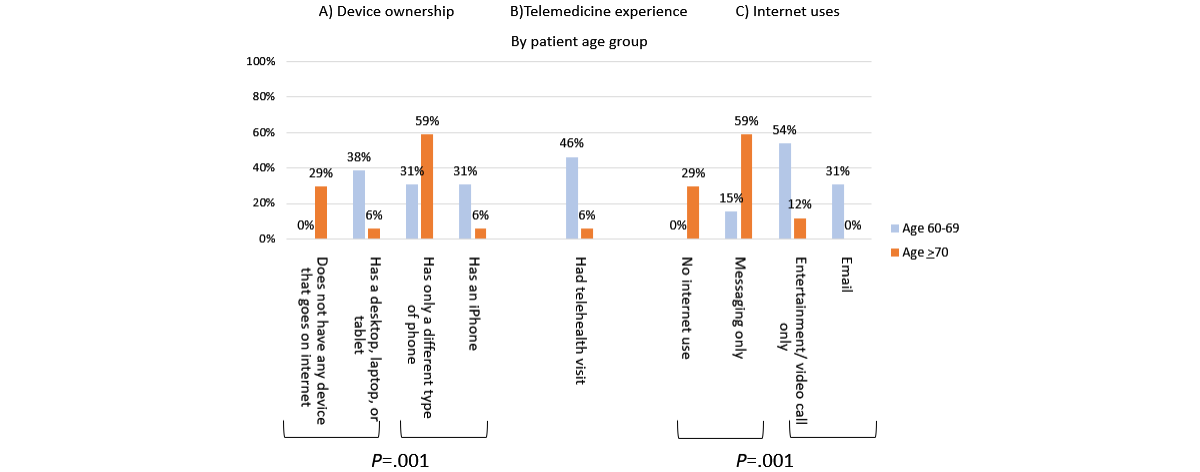
Comparison of device ownership, telemedicine experience, and internet uses by age group.

Of 30 respondents, 7 (23%) had had a telemedicine appointment. Participants who owned a computer or iPhone were more likely to have had a telemedicine visit than others (Figure 1A; *χ^2^*_1_=9.5; *P*=.002), as were participants who had used the internet for email or functions other than messaging (Figure 1B; *χ^2^*_1_=11.9; *P*<.001). All but one respondent who had a telemedicine visit had an iPhone or a computer and used internet functions other than messaging. Participants with iPhones or computers used their devices for a broader range of tasks (Table 2; *χ^2^*_3_=18.0; *P*<.001), endorsed fewer telemedicine disadvantages (*χ^2^*_3_=11.9; *P*=.008), and were more likely to indicate interest in future telemedicine visits (*χ^2^*_1_=5.7; *P*=.02) than were patients with other types of mobile devices or no devices at all. Telemedicine attitudes of patients who used email or other internet functions were similar to those with advanced devices. Loss of connection with their doctor was the most commonly endorsed telemedicine disadvantage (10/30, 33%) followed by concerns about exam privacy and quality (7/30, 23%). Patients who were aged 65-70 years were more likely to have an iPhone or other computer (*χ^2^*_1_=10.5; *P*=.001; Figure 2A), and were more likely to have had a telemedicine visit (*χ^2^_1_*=6.7; *P*=.01; Figure 2B) and to have used internet functions other than messaging (*χ^2^*_1_=15.9; *P*<.001; Figure 2C) compared with patients aged 70 years and older.

**Table 2:** Internet uses and telemedicine attitudes by device type.

Devices and functions used		Internet uses			Telemedicine attitudes	
		Mean number of ways participants use the internet	Messaging only, n (%)	Mean number of telemedicine disadvantages		Interest in future telemedicine visit, n (%)
**Type of device**						
	iPhone	2.4	0 (0)	0.4		5 (100)
	iPad or computer	2.3	1 (8)	0.7		4 (67)
	Other mobile only	1.2	11 (92)	1.4		6 (43)
	None	0.0	0 (0)	1.2		1 (20)
**Internet functions used**						
	Used email	N/A^a^	N/A	0.5		4 (100)
	No email but used entertainment or video calling	N/A	N/A	0.7		7 (77.8)
	Used messaging only	N/A	N/A	1.4		4 (33.3)
	No internet use	N/A	N/A	1.2		1 (20)

^a^N/A: not applicable.

## Discussion

### Principal Findings

This small survey revealed significant gaps in telemedicine readiness among older adults who said they had devices that could be used for telemedicine and that they went online. No patients used key internet functions needed for staying safe during the COVID-19 pandemic, and few used internet applications that required the skills needed for accessing telemedicine. Few patients had devices that are optimal for older adults using telemedicine. Patients with more advanced devices used more internet functions and had more telemedicine experience and more favorable attitudes than others. Our results confirm previous studies [10-12] showing generally lower technological proficiency among older adults and some concerns about participating in telemedicine. However, our study is novel in pointing to subtle dimensions of telemedicine readiness that warrant further study—device capacity and use of internet in ways that build skills needed for telemedicine such as email and video calling. Before training older adults to use telemedicine, it is important to ensure that they have the devices, basic digital skills, and connectivity needed for telemedicine. Screening for readiness may require nuanced assessment regarding specific device capacity and skills.

### Limitations and Future Directions

Because of the survey’s limited nature, other important topics, such as home internet access and interest in digital skills training, could not be addressed. Results may not be generalizable to other contexts, such as specialty clinics or rural areas. Participants present in the clinic may be different from those not seeking care, which could bias our results. Larger studies are needed to confirm our results and apply multivariate analysis to understand the relationships among age, device quality, internet skills, and telemedicine attitudes. Development of validated scales of telemedicine readiness as well as telemedicine training to complement in-person care can help health systems offer precision-matched interventions to address barriers, facilitate increased adoption, and generally improve patients’ overall access to primary care and engagement with their primary care provider.

## Appendix

Multimedia Appendix 1 Survey questions.
