# Supplementary figures and images for: Telemedicine Preparedness Among Older Adults With Chronic Illness: Survey of Primary Care Patients
Source: JMIR Form Res. 2022 Jul 27;6(7):e35028. doi: 10.2196/35028 (PMC9377459; doi:10.2196/35028)

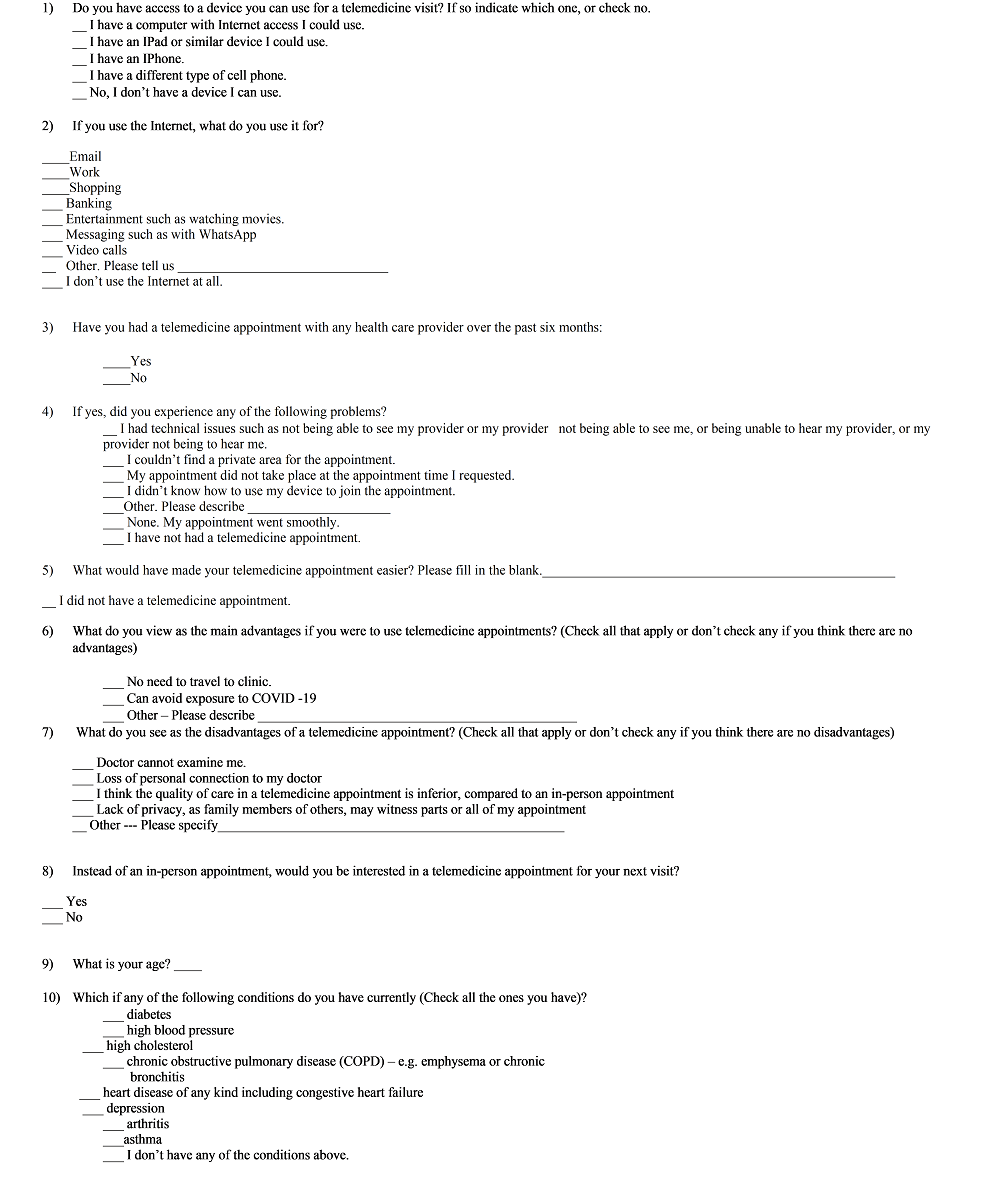

Supplement: Multimedia Appendix 1 [file formative_v6i7e35028_app1.png]
